# ﻿Chromosomes of four fishfly species (Megaloptera, Corydalidae, Chauliodinae) from North America

**DOI:** 10.3897/compcytogen.19.146136

**Published:** 2025-04-16

**Authors:** Yoshinori Takeuchi, Koji Iizuka, Tadashi Nakazato, Hiroyuki Koishi, Hidehiro Hoshiba

**Affiliations:** 1 Bohkai Junior High School, 1-1-33 Nishi-Akashi, Akashi, Hyogo 673-0041, Japan Bohkai Junior High School Hyogo Japan; 2 Matsue 4th Junior High School, 1-16-1 Nishi-Ichinoe, Edogawa-ku, Tokyo 132-0023, Japan Matsue 4th Junior High School Tokyo Japan; 3 Shikinejima Junior High School, 166 Shikinejima, Niijimason, Tokyo 111-0511, Japan hikinejima Junior High School Tokyo Japan; 4 College of Agriculture, Tamagawa Univesity, 1-1 Tamagawagakuen, Machida, Tokyo 194-8610, Japan Tamagawa Univesity Tokyo Japan

**Keywords:** Chromosomes, *Dysmicohermes*, fishflies, *Nigronia*, *Orohermes*, sex chromosomes, Xy_p_/XX

## Abstract

We analyzed chromosomes of four species of fishflies (Megaloptera: Chauliodinae). Three species were from western North America (*Dysmicohermesdisjunctus* (Walker, 1866), *Dysmicohermesingens* Chandler, 1954, and *Orohermescrepusculus* (Chandler, 1954)), and another one from eastern North America (*Nigroniaserricornis* (Say, 1824)). The chromosome number of the three western species was 2n = 22, with the karyotype consisting of 10 pairs of autosomes plus XY in males. The X chromosomes of these three species are subtelocentric, while the Y chromosomes are small and dot-like. Of the ten pairs of autosomes, the last pair is substantially smaller than the others. The chromosome number in the first meiotic metaphase in spermatocytes of *N.serricornis* from Michigan was n = 10 (9 autosomal bivalents + Xy_p_ in the male). The sex chromosomes of *N.serricornis* formed parachute-type bivalents synchronously with the autosomes. The parachute-type bivalent Xy_p_ has also been found in four fishflies and four dobsonflies (Megaloptera: Corydalinae) from East Asia, as well as in a fishfly and a dobsonfly from North America. These data suggest that the two subfamilies of Corydalidae share a common sex-bivalent mechanism, along with many beetles (Coleoptera).

## ﻿Introduction

The insect order Megaloptera comprises the families Sialidae (alderflies) and Corydalidae. The latter contains the two subfamilies, Corydalinae (dobsonflies) and Chauliodinae (fishflies). In North America 49 species of Megaloptera are recognized, including 24 Sialidae, 6 Corydalinae, and 19 Chauliodinae (Martins et al. 2022). The larvae of all Megaloptera are aquatic and predaceous, and they perform important functions in aquatic food webs (Rivera-Gasperin et al. 2019). Corydalinae larvae inhabit deeper waters in streams and rivers and absorb oxygen through tracheal gills on the ventral surface of their abdomens. By contrast, Chauliodinae larvae live near the banks of rivers and are primarily air breathers, using a pair of respiratory tubes on the dorsal surface of the abdomen.

Studies on Megaloptera chromosomes have been reported by Itoh (1933a, b), Hughes-Schrader (1980), and Takeuchi et al. (2002, 2012a, b). Four Japanese species of Chauliodinae had the chromosome number 2n = 20 (18+XY) (Takeuchi et al. 2012a, b) and one species from North America had the chromosome number 2n = 22 (20+XY) (Hughes-Schrader 1980). On the other hand two species of Corydalinae, from Japan and North America, both had the chromosome number 2n = 24 (22 + XY) (Hughes-Schrader 1980; Takeuchi et al. 2002).

In the present study, chromosomes of four fishfly species from the United States were studied, using preparations from larval gonads. These included three western species [*Dysmicohermesdisjunctus* (Walker, 1866), *Dysmicohermesingens* Chandler, 1954 and *Orohermescrepusculus* (Chandler, 1954)], and one eastern species [*Nigroniaserricornis* (Say, 1824)] (Lacewing Digital Library 2025). Life-history information on these four fishfly species can be found in Evans (1972) and Heilveil (2004). According to data in Lacewing Digital Library (2025), there is only one species in the genus *Orohermes* in the world fauna, and each of the genera *Dysmicohermes* and *Nigronia* harbors two species. The objective of the current study was to characterize the chromosomes of the above four North American Chauliodinae species and to compare the results with East Asian Chauliodinae as well as several Corydalinae species. We also discuss the results in terms of insect evolutionary history.

## ﻿Material and methods

### ﻿Insects

Final-instar larvae of *D.disjunctus*, *D.ingens*, and *O.crepusculus* were collected along the banks of creeks and rivers in Oregon and California during August 1997. Similarly, final-instar larvae of *N.serricornis* were collected during August 2002 in Michigan. Collection sites, sampling dates, and the numbers of larvae studied are given in Table 1. In the field, larvae were placed individually in cups, filled with water and transported back to Japan alive. The larvae were fed with aquatic insects before dissection. Ten nuclei from spermatogonia from two males were examined for each of the three western species. Similarly, ten spermatocytes from two males were examined for the eastern species *N.serricornis*. The mitotic karyotypes were described following the nomenclature suggested by Levan et al. (1964). Since mitotic and meiotic divisions were not obtained from females, only male specimens were used.

**Table 1.** Material used. Collection sites, sampling dates, and number of studied final-instar male larvae of four fishflies species.

Taxon	Sampling locality and date of collection	No. of studied larvae
*Orohermescrepusculus*	USA, Oregon, Vida, Gate Creek, a tributary of the McKenzie River (altitude: about 180 m); 44.1467°N, 122.5735°W; August, 1997	2
*Dysmicohermesdisjunctus*	USA, Oregon, Flynn, Dinner Creek at the foot of Mt. Marys Peak (altitude: about 700 m); 44.4765°N, 123.5037°W; August, 1997	2
*Dysmicohermesingens*	USA, California, North Placerville, a small Creek that is a tributary of South Fork American River (altitude: about 380 m); 38.7676°N, 120.8060°W; August, 1997	2
*Nigroniaserricornis*	USA, Michigan, Williamston, Red Cedar River; 42.6912°N, 84.2846°W; August, 2002	2

### ﻿Chromosome preparation

For all four species the gonads of the final-instar larvae were studied. Dissections were conducted during December 1997 for the three western species and during December 2002 for the eastern species. Preparations were made by the lactic acid dissociation, and drying method (Hoshiba et al. 1989). The tissues were dissected from the larvae in 1% sodium citrate and then placed in a colchicin solution (0.005%) for 30 minutes. The tissues were then placed on individual clean glass slides, after which three drops of a fixative I (F1: 3 parts glacial acetic acid, 3 parts of 99% ethanol, 4 parts of water) were added using a pipette. Next, a tiny drop of dissociation solution (1 part of 30% lactic acid diluted in glacial acetic acid and 2 or 3 parts of F1) was put on the tissue. The tissue samples were dissociated using a needle for 10–15 seconds. Then, two drops of a second fixative (F2: 1 part glacial acetic acid: 1part 99% ethanol) were added to the cells by pipette, after which excess fixative and dissociation solution were removed with filter paper. Each slide was then dried, but just before the slide was completely dry, a drop of glacial acetic acid was added to the cells. To complete drying, the slides were then placed in an incubator overnight at 40–50 °C.

### ﻿Chromosome staining

The chromosomes were stained with 3% Giemsa’s solution in Sorensen’s phosphate buffer at pH 6.8 for 20 min (Hoshiba et al. 1989).

### ﻿Microscopy and imaging

Microscopic photography of the chromosomal preparations was performed using an optical microscope (OL-IM) connected to a Microflex Afx-dx (both manufactured by Japan Optical Industry Co., Ltd.). Photographs of selected chromosome spreads were made using a 100× oil immersion objective. Photographs were taken using Mini-copy film ISO25 and Sencia ISO100 (both manufactured by Fujifilm Co., Ltd.) and printed on Fuji WP FM2~3 photographic paper.

## ﻿Results

**Figure 1.**
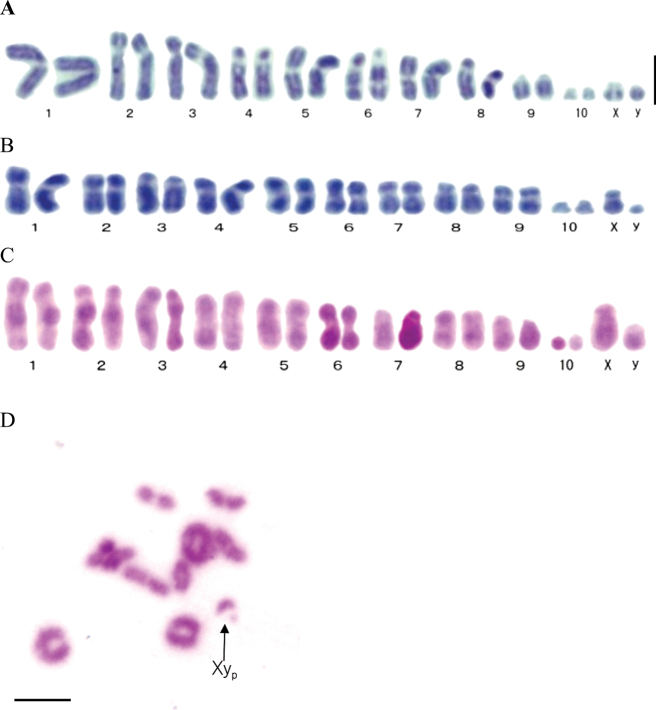
Chromosomes of fishflies. Mitotic karyotypes of spermatogonial cells (**A–C**) and diakinesis of meiosis in the spermatocyte (**D**) **A***Orohermescrepusculus* (2n = 20 + XY) **B***Dysmicohermesdisjunctus* (2n = 20 + XY) **C***Dysmicohermesingens* (male: 2n = 20 + XY) **D***Nigroniaserricornis* (2n = 18 + Xy_p_). Arrow indicates the parachute-type sex bivalent (Xy_p_). Scale bar: 5 μm.

In the present study, the chromosome number of 2n = 22 was found in the three species from western North America (10 autosomal pairs + XY in males) (Fig. 1A–C). The autosomes of *O.crepusculus* included four pairs of metacentric (no. 1, 5, 7, and 8), five pairs of submetacentric (no. 2, 3, 4, 6, and 9), and a single pair of small subtelocentric chromosomes (no. 10). The sex chromosomes were represented by the small subtelocentric X chromosome and the small, almost dot-like, Y chromosome (Fig. 1A). Secondary constrictions were seen near the center of the long arm in chromosomes no. 4 and 6 (Fig. 1A). The autosomes of *D.disjunctus* included six pairs of metacentric (no. 1, 4, 5, 6, 7, and 9), three pairs of submetacentric (no. 2, 3, and 8), and a single pair of small subtelocentric chromosomes (no. 10). The sex chromosomes were represented by the smaller subtelocentric X chromosome and the small dot-like Y chromosome (Fig. 1B). The autosomes of *D.ingens* included three pairs of metacentric (no. 4, 6, and 8), four pairs of submetacentric (no. 1, 2, 3, and 5) and three pairs of subtelocentric (no. 7, 9, and 10), with no. 10 being relatively small. The sex chromosomes were represented by the medium-sized subtelocentric X chromosome and the smaller dot-like subtelocentric Y chromosome (Fig. 1C). The chromosome number at the first meiotic metaphase (MI) in spermatocytes of *N.serricornis* was n = 10 (9 autosomal bivalents + Xy_p_ in the male) (Fig. 1D). During this division, sex chromosomes invariably formed parachute-type bivalents synchronously with the autosomes (Fig. 1D).

## ﻿Discussion

Hughes-Shrader (1980) observed a single pair of small autosomes in the fishfly *Neohermesfillicornis* (Banks, 1903), which is native to western North America (Table 2). In the present study, the three western fishfly species also had one pair of small autosomes (Fig. 1A–C, Table 2). By contrast, in four Asian fishfly species, as well as in the species from Michigan, the pair of small autosomes is lacking and they all have a chromosome number of 2n = 20 (9 autosomal pairs/bivalents + Xy_p_ in the male) (Table 2, Takeuchi et al. 2012a, b). In addition, in five dobsonfly species from East Asia and the United States, all have a single pair of dot-like autosomes (Table 2). The presence or absence of this characteristic chromosome pair might play an important role in the karyotype evolution in the Corydalidae, which consists of Corydalinae (dobsonflies) and Chauliodinae (fishflies). In terms of the chromosome number, both East Asian and American dobsonflies have 2n = 24 (22 + Xy_p_ in the male), but in fishflies, 2n = 20 (18 + Xy_p_ in the male) in East Asian species and n = 10 (9 + Xy_p_ in the male) in a Michigan species (Table 2). These findings indicate that the chromosome number and karyotype structure are more variable in fishflies than in dobsonflies.

**Table 2.** Chromosome numbers of species of Corydalidae (Megaloptera) so far studies with karyotype descriptions. LM: large metacentric; LSM: large submetacentric; M: metacentric; SM: submetacentric; ST: subtelocentric; T: telocentric; dot: a very small chromosome.

Family or Subfamily Species	Chromosome number (2n)	Morphology				Method	Distribution	Authors
		Autosomes	X	Y	Meiosis (sex chromosome)			
Corydalinae (Dobsonflies)								
*Protohermesgrandis*	24	1LSM+2M+7T+1dot	SM	dot	Xy_p_	drying-1*	Japan (Honsyuh)	Takeuchi et al. 2002
*Protohermesimmaculatus*	24	1LSM+2M+2ST+5T+1dot	SM	dot	Xy_p_	drying-2**	Japan (Amami***)	Takeuchi et al. Preparation
*Protohermesdisjunctus*	24	1LSM+9T+1dot	SM	dot	Xy_p_	drying-2**	Japan (Ishigaki****)	Takeuchi et al. Preparation
*Protohermescostalis*	24	1LSM+9T+1dot	SM	dot	Xy_p_	drying-2**	Taiwan	Takeuchi et al. Preparation
*Corydaluscornutus*	24	1LM+1M+8T+1dot	SM	dot	Xy_p_	squash	U.S.A (North Carolina)	Hughes-Schrader 1980
Chauliodinae (Fishflies)								
*Parachauliodescontinentalis*	20	1LSM+1M+7T	ST	dot	Xy_p_	drying-2**	Japan (Honsyuh)	Takeuchi et al. 2012a
*Parachauliodesjaponicus*	20	1LSM+1SM+4M+3ST	ST	dot	Xy_p_	drying-2**	Japan (Honsyuh)	Takeuchi et al. 2012a
*Neochauliodesnigris*	20	4M+1SM+4ST	M	dot	Xy_p_	drying-2**	Japan (Ishigaki****)	Takeuchi et al. 2012b
*Neochauliodesformosanus*	20	4M+5ST	SM	dot	Xy_p_	drying-2**	Taiwan	Takeuchi et al. 2012b
*Orohermescrepusculus*	22	4M+5SM+1smallST	ST	dot	–	drying-2**	U.S.A (Oregon)	present study
*Dysmicohermesdisjunctus*	22	6M+3SM+1smallST	ST	dot	–	drying-2**	U.S.A (Oregon)	present study
*Dysmicohermesingens*	22	3M+4SM+3ST+1smallT	ST	small	–	drying-2**	U.S.A (California)	present study
*Nigroniaserricornis*	20	–	–	dot	Xy_p_	drying-2**	U.S.A (Michigan)	present study
*Neohermesfillicornis*	22	10M+1smallST	M	dot	Xy_p_	squash	U.S.A (California)	Hughes-Schrader 1980

* Method by Kezer and Sessions (1979); ** Method by Hoshiba et al. (1989); *** Amami-Oshima Island; **** Ishigaki Island

The secondary constrictions of chromosomes no. 4 and 6 in *O.crepusculus* (Fig. 1A) suggest that they may be the sites of rDNA clusters. In other words, the sites of rDNA clusters are observed in the secondary constrictions of chromosomes and are often located at nucleolus organizer regions (NOR). Future verification using techniques such as FISH (fluorescence in situ hybridization) or AgNOR (silver stain for nucleolus organizer region) is required (Iizuka et al. 2013; Kuznetsova et al. 2016).

In Megaloptera, the Y chromosome is dot-shaped, and when pairing occurs with the X, the XY approach each other as in autosomes, so they appear parachute-shaped. The parachute-type bivalent Xy_p_ was detected in *N.serricornis* in the present study (Fig. 1D). The parachute-type bivalent Xy_p_ has also been found in four other fishfly and four dobsonfly species from East Asia (Takeuchi et al. 2002, 2012a, b) (Table 2), as well as in the North American dobsonfly *Corydaluscornutus* Linnaeus, 1758 and fishfly *N.fillicornis* (Hughes-Shrader, 1980). Therefore, species of both subfamilies Corydalidae share a common sex-bivalent mechanism.

Megaloptera was once included in the order Neuroptera, but today is considered an independent order (Hayashi, 2018). The sex chromosomes in antlions and owlflies (both Neuroptera) are of the XY type (Kuznetsova et al. 2015). However, the parachute-type bivalent Xy_p_ has not been found in current members of the Neuroptera. The Xy_p_ configuration of meiotic sex chromosomes (Smith 1950) is found in many beetle (Coleoptera) families (Petitpierre 2011; Şendoğan and Alpagut-Keskin 2016; Okutaner 2020; Ruiz-Torres et al. 2021). Interestingly, recent phylogenetic analyses of DNA sequence data recognize the Coleoptera as a sister group of Megaloptera (Beutel et al. 2018). This close relationship between Megaloptera and Coleoptera is further corroborated by the presence of the parachute-type bivalent Xy_p_ in both orders.
